# Comparative Genome Analysis of an Extensively Drug-Resistant Isolate of Avian Sequence Type 167 *Escherichia coli* Strain Sanji with Novel *In Silico* Serotype O89b:H9

**DOI:** 10.1128/mSystems.00242-18

**Published:** 2019-02-26

**Authors:** Xiancheng Zeng, Xuelin Chi, Brian T. Ho, Damee Moon, Christine Lambert, Richard J. Hall, Primo Baybayan, Shihua Wang, Brenda A. Wilson, Mengfei Ho

**Affiliations:** a Key Laboratory of Fujian-Taiwan Animal Pathogen Biology, College of Animal Sciences, Fujian Agriculture and Forestry University, Fuzhou, China; b College of Life Sciences, Fujian Agriculture and Forestry University, Fuzhou, China; c Department of Microbiology and Molecular Genetics, Harvard Medical School, Boston, Massachusetts, USA; d Department of Microbiology, School of Molecular and Cellular Biology, University of Illinois at Urbana-Champaign, Urbana, Illinois, USA; e Pacific Biosciences, Menlo Park, California, USA; University of Trento

**Keywords:** O-antigen, antibiotic resistance, capsular polysaccharide, extensively drug resistant, genome comparison, insertion sequence, pathogen evolution, plasmid-mediated resistance, prophage, secretion systems

## Abstract

Extensive drug resistance (XDR) is an escalating global problem. Escherichia coli strain Sanji was isolated from an outbreak of pheasant colibacillosis in Fujian province, China, in 2011. This strain has XDR properties, exhibiting sensitivity to carbapenems but no other classes of known antibiotics. Whole-genome sequencing revealed a total of 32 known antibiotic resistance genes, many associated with insertion sequence 26 (IS*26*) elements. These were found on the Sanji chromosome and 2 of its 6 plasmids, pSJ_255 and pSJ_82. The Sanji chromosome also harbors a type 2 secretion system (T2SS), a type 3 secretion system (T3SS), a type 6 secretion system (T6SS), and several putative prophages. Sanji and other ST167 strains have a previously uncharacterized O-antigen (O89b) that is most closely related to serotype O89 as determined on the basis of analysis of the *wzm-wzt* genes and *in silico* serotyping. This O89b-antigen gene cluster was also found in the genomes of a few other pathogenic sequence type 617 (ST617) and ST10 complex strains. A time-scaled phylogeny inferred from comparative single nucleotide variant analysis indicated that development of these O89b-containing lineages emerged about 30 years ago. Comparative sequence analysis revealed that the core genome of Sanji is nearly identical to that of several recently sequenced strains of pathogenic XDR E. coli belonging to the ST167 group. Comparison of the mobile elements among the different ST167 genomes revealed that each genome carries a distinct set of multidrug resistance genes on different types of plasmids, indicating that there are multiple paths toward the emergence of XDR in E. coli.

**IMPORTANCE** E. coli strain Sanji is the first sequenced and analyzed genome of the recently emerged pathogenic XDR strains with sequence type ST167 and novel *in silico* serotype O89b:H9. Comparison of the genomes of Sanji with other ST167 strains revealed distinct sets of different plasmids, mobile IS elements, and antibiotic resistance genes in each genome, indicating that there exist multiple paths toward achieving XDR. The emergence of these pathogenic ST167 E. coli strains with diverse XDR capabilities highlights the difficulty of preventing or mitigating the development of XDR properties in bacteria and points to the importance of better understanding of the shared underlying virulence mechanisms and physiology of pathogenic bacteria.

**Author Video**: An author video summary of this article is available.

## Abstract (precis)

E. coli strain Sanji is the first sequenced and analyzed genome of the recently emerged pathogenic XDR strains with sequence type ST167 and novel *in silico* serotype O89b:H9. Comparison of the genomes of Sanji with other ST167 strains revealed distinct sets of different plasmids, mobile IS elements, and antibiotic resistance genes in each genome, indicating that there exist multiple paths toward achieving XDR. The emergence of these pathogenic ST167 E. coli strains with diverse XDR capabilities highlights the difficulty of preventing or mitigating the development of XDR properties in bacteria and points to the importance of better understanding of the shared underlying virulence mechanisms and physiology of pathogenic bacteria.

## INTRODUCTION

The alarming increase in multidrug-resistant (MDR) and extensively drug-resistant (XDR) bacterial strains is a global health crisis (1–3). Many currently circulating intestinal pathogenic Escherichia coli strains, such as the well-known O157:H7 strain (4, 5), are still susceptible to antibiotics. However, the threat of pathogenic E. coli acquiring antibiotic resistance genes from environmental reservoirs is of escalating concern (6, 7), and more-recent O104:H4 clonal lineages have acquired not only Shiga toxin-encoding phage but also extended-spectrum-β-lactamase (ESBL) resistance (8, 9). To tackle this problem, it is important to understand not only how multiple antibiotic resistances are acquired but also how they can be accumulated within a commensal or pathogenic bacterium.

Certain traits of genomes in transition toward niche or host adaptation include an increase in mobile genetic elements that imbue the bacteria with the potential to acquire additional traits that might enhance virulence in the host (10). Mobile genetic elements, such as plasmids, bacteriophages, insertion sequence (IS) elements, and transposons, are well-established players in the acquisition of virulence traits leading to the emergence and evolution of bacterial pathogens. Despite the critical role that plasmids and other mobile genetic elements play in antibiotic resistance spread (11, 12), we still cannot predict which resistance genes or plasmids will be acquired by a bacterial pathogen to cause the next XDR superbug to emerge.

Comparative whole-genome sequence analysis of MDR/XDR strains has enabled phylogenetic studies into the evolutionary mechanisms involved in acquisition and accumulation of antibiotic resistance genes (12), including studies exploring evolutionary trade-offs between virulence and resistance (13–16); tracking the spread of resistant pathogens (17–19), or monitoring within-host evolution of pathogens (20, 21). One comparative genomics study revealed the stepwise evolutionary process by which a highly infectious clone of extraintestinal pathogenic E. coli (ExPEC) of sequence type 131 (ST131) gained multiple virulence and antibiotic resistance gene clusters over a period of about 60 years (22), ultimately leading to its current global dominance as an XDR pathogen (23). A similar pattern of sequential emergence of increasing virulence potential and antibiotic resistances over a period of 30 years has been documented for another pathogenic E. coli clonal group, ST393 (24).

We report the comparative genome characterization of pathogenic E. coli strain Sanji, which was isolated from pheasants during a 2011 outbreak of colibacillosis and was refractory to clinical application of commonly used veterinary antibiotics. Antibiotic susceptibility testing confirmed that the isolate was XDR. Whole-genome sequencing of the bacterial genome, including its six plasmids, and comparative multilocus sequence typing (MLST) revealed that the core genome of Sanji is nearly identical to the genomes of a number of recently sequenced pathogenic XDR E. coli strains belonging to sequence type ST167. *In silico* serotyping revealed that Sanji, like other ST167 strains, has a unique capsular polysaccharide gene cluster and a previously unidentified *in silico* serotype, O89b. The presence of numerous antibiotic resistance gene clusters and IS*26* elements accounts for the observed XDR phenotype. Comparison of Sanji to other members of the ST167 lineage further revealed the extent and diversity of the paths used by these bacteria to achieve XDR. This group of ST167 strains represents another emerging pathogenic clonal lineage with XDR.

## RESULTS AND DISCUSSION

### Antibiotic susceptibility profile of E. coli Sanji.

**FIG 1:**
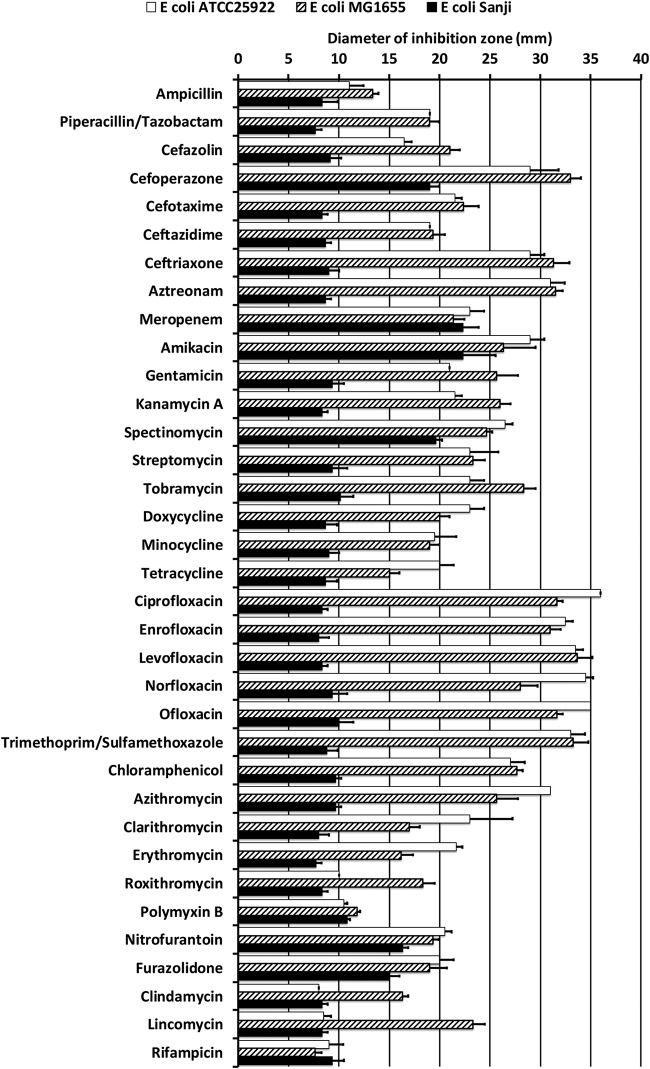
Antibiotic susceptibility profiles for E. coli strains Sanji, MG1655, and ATCC 25922. Shown are the mean zones of inhibition (in millimeters) recorded for Kirby-Bauer disc diffusion assays (6.5-mm to 7.0-mm disc diameter) for the indicated antibiotics. Open bars, E. coli ATCC 25922; hatched bars, E. coli MG1655; black bars, E. coli Sanji. Error bars represent means ± standard deviations of results from three independent experiments. Direct comparison of Sanji with ATCC 25922 and MG1655 showed little or no susceptibility of Sanji to most of the antibiotics listed (black bars), as evidenced by the lack of a zone of inhibition beyond the disk diameter. Note that Sanji and MG1655 were found to be susceptible to polymyxin B and colistin by the broth microdilution method.

The antibiotic susceptibility profile of E. coli Sanji was compared directly to that of two reference strains: E. coli ATCC 25922, a standard strain used by the CLSI, and E. coli MG1655, a prototype K-12 strain chosen for its genetic similarity to E. coli Sanji. As shown in Fig. 1, Sanji has resistance to most antibiotics, exhibiting sensitivity only to carbapenem (meropenem) and partial sensitivity to a few others (e.g., amikacin, spectinomycin, furazolidone, and nitrofurantoin). Sanji also exhibits resistance to a β-lactam combination with β-lactamase inhibitor (piperacillin-tazobactam). All three E. coli strains, Sanji, MG1655, and ATCC 25922, displayed apparent resistance in the Kirby-Bauer assay to polymyxin B, even though they do not possess the *mcr-1* gene. Sanji does possess a phosphoethanolamine transferase (*eptA*) gene with homology to all *mcr* genes, notably, 41% identity with *mcr-3* and 43% identity with *mcr-8*. However, this *eptA* gene is also present in MG1655 and many other E. coli strains. When tested against polymyxin B and colistin using the broth microdilution method, the observed MICs for Sanji (0.3 µg/ml each for colistin and polymyxin B) were only 2-fold higher than that of MG1655 and not 10-fold to 100-fold higher (MIC of 3 to 32 µg/ml) such as would be expected for *mcr-1*-mediated resistance (25).

### Antibiotic resistance genes in E. coli Sanji.

**FIG 2:**
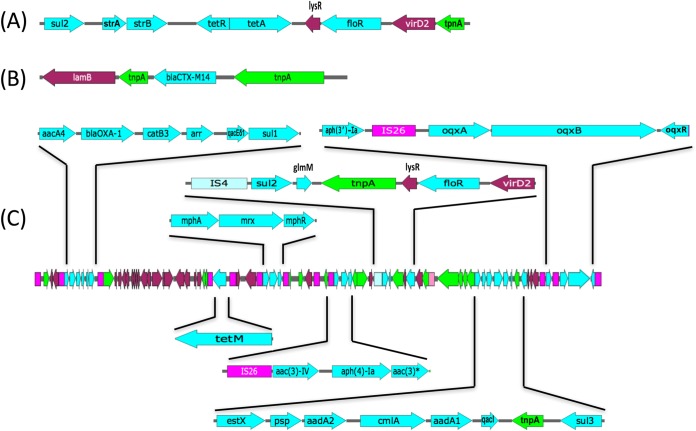
Antibiotic resistance gene clusters in E. coli Sanji. (A) An 8.9-kb resistance gene cluster was found in the 69.2-kb insertion on the chromosome. (B) *blaCTX-M14* gene plus flanking genes found on plasmid pSJ_82. (C) An 80-kb resistance gene cluster was found on plasmid pSJ_255. In panels A to C, known antibiotic resistance genes are indicated in cyan; IS*26* elements in magenta; IS*4* elements in pale blue; IS*1006* elements in pink; transposase genes in green; and other genes in maroon. The asterisk denotes a gene with a GNAT domain that overlaps a transposase gene.

PacBio whole-genome sequencing revealed that Sanji consists of a 4.9-Mb chromosome and 6 plasmids: pSJ_255 (255.4-kb), pSJ_98 (98.4-kb), pSJ_94 (94.7-kb), pSJ_82 (82.3-kb), pSJ_3 (3.4-kb), and pSJ_2 (2.6-kb). Sanji has all of the known drug efflux pump genes belonging to all five classes of drug transporters found in MG1655 (26). The Sanji chromosome harbors an 8.9-kb cluster of genes associated with known drug resistance to sulfonamides (*sul2*), aminoglycosides (*strAB*), tetracycline (*tetRA*), and chloramphenicol (*floR*) (Fig. 2A). In addition to this locus, we identified a total of 32 distinct antibiotic resistance genes in Sanji within identifiable mobile elements (Table 1), including 6 genes within the chromosome, 1 gene on plasmid pSJ_82, and 27 genes on the large plasmid, pSJ_255, with two of the genes appearing in both the chromosome and a plasmid. The resistance gene identified on pSJ_82 encodes a class A extended-spectrum β-lactamase (ESBL), *blaCTX-M-14* (Fig. 2B). CTX-M ESBLs have been implicated in resistance to third-generation β-lactams in multiple *Enterobacteriaceae* species (27). All 27 of the antibiotic resistance genes on pSJ_255 were localized to an 80-kb region (Fig. 2C). The genes carried on pSJ_255 included those conferring resistance to β-lactams (*blaOXA-1*), tetracyclines (*tetM*), aminoglycosides [*aac(6')-Ib*, *aac(3)-IVa*, *aac(4)-Ia*, *aadA2*, *aadA1*, *aph(3′)-Ia*, *aph(*4*)-Ia*, and *aac(3)*], chloramphenicol (*catB3*, *floR*, and *cmlA1*), rifampin (*arr*), quaternary ammonium compounds (*qacEδ1* and *qacI*), sulfonamides (*sul1*, *sul2*, and *sul3*), and macrolides (*mphA*, *mrx*, *mphR*, and *glmM*), as well as a known RND multidrug efflux pump (*oqxABR*).

**TABLE 1:** Antibiotic resistance genes found in ST167 Escherichia coli strains^*a*^

Parameter	Result for E. coli strain:														
	Sanji	ECONIH6	AR_0011	AR_0014	AR_0149	AR_0150	AR_0151	AR_0162	WCHEC005237	FDAARGOS_434	CRE1493	CREC-532	CREC-629	Y5	SCEC020007
No. of plasmids	6	2	3	2	2	3	2	4	8	1	5	3	3	3	2
Total no. of AR genes^*b*^	32 (34)	21 (22)	12 (13)	8	3	14	4	15	21 (28)	15 (24)	30 (36)	19 (25)	19 (23)	25 (43)	16 (24)
Chromosome-carried AR genes	6	0	1	0	0	0	0	0	0	0	0	6	6	12 (20)	0
Plasmid-carried AR genes	28	21 (22)	12 (13)	8	3	14	4	15	21 (28)	15 (24)	30 (36)	17 (19)	17	21 (23)	16 (24)
No. of IS*26* elements on plasmids	12	8	6	6	1	3	1	9	10	5	12	10	8	10	6
No. of AR genes near IS*26*^*c*^	24	16	12	8	0	12	2	13	8	24	25	15	8	21	22

Presence of gene:															
*aac(3)-IIa*	N	N	Y	Y	N	N	N	N	N	N	Y	N	N	N	N
*aac(3)-IId*	N	N	N	N	N	N	N	N	N	N	Y	Y	Y	Y	N
*aac(3)-IVa*	Y	N	N	N	N	N	N	N	N	N	N	N	N	N	N
aac(3)*^d^	Y	N	N	N	N	N	N	N	N	N	N	N	N	N	N
*aac(6')Ib-cr*	Y	N	Y	Y	N	N	N	N	Y	N	Y	N	N	Y	N
*aadA1*	Y	N	N	N	N	N	N	N	N	N	N	N	N	N	N
*aadA2*	Y	Y	N	N	N	N	N	N	N	Y	Y	N	N	N	N
*aadA5*	N	N	N	N	N	Y	N	N	N	Y	Y	Y	Y	Y	Y
*aadA16*	N	N	N	N	N	N	N	N	Y	N	N	N	N	N	Y
*aph(4)-Ia*	Y	N	N	N	N	N	N	N	N	N	N	N	N	N	N
*aph(3′)-Ia*	Y	N	N	N	N	N	N	N	N	N	N	N	N	Y	N
*arr-3*	Y	N	N	N	N	N	N	N	Y	N	N	N	N	Y	N
*blaCMY-42*	N	N	N	N	Y	Y	Y	N	N	N	N	N	N	Y	N
*blaCTX-M-14*	Y	N	N	N	N	N	N	N	N	N	N	Y	Y	Y	N
*blaCTX-M-15*	N	Y	Y	Y	N	N	N	Y	N	Y	Y	N	N	Y	N
*blaCTX-M-55*	N	N	N	N	N	N	N	N	Y	N	N	Y	Y	N	N
*blaNDM-5*	N	Y	N	N	N	Y	Y	N	Y	Y	Y	N	N	N	Y
*blaNDM-7*	N	N	N	N	Y	N	N	Y	N	N	N	Y	Y	Y	N
*blaOXA-1*	Y	Y	Y	Y	N	N	N	N	N	N	Y	N	N	Y	N
*blaTEM-1A*	N	N	N	N	N	N	N	N	N	N	Y	N	N	N	N
*blaTEM-1B*	N	N	N	N	N	Y	Y	Y	Y	N	N	Y	Y	N	Y
*ble*	N	Y	N	N	Y	Y	Y	Y	Y	Y	Y	Y	Y	Y	Y
*catB3*	Y	N	N	N	N	N	N	N	N	N	N	N	N	N	N
*cmlA1*	Y	N	N	N	N	N	N	N	N	N	N	N	N	N	N
*dfrA12*	N	Y	N	N	N	N	N	N	N	Y	Y	N	N	N	Y
*dfrA14*	N	Y	N	N	N	N	N	N	Y	N	N	N	N	N	N
*dfrA17*	N	N	N	N	N	Y	N	N	N	Y	Y	Y	Y	Y	Y
*dfrA27*	N	N	N	N	N	N	N	N	Y	N	N	N	N	Y	N
*eamA*	N	Y	Y	Y	N	Y	N	Y	Y	Y	Y	N	N	Y	Y
*erm*(B)	N	Y	N	N	N	N	N	Y	N	N	N	Y	Y	N	N
*estX*	Y	N	N	N	N	N	N	N	N	N	N	N	N	N	N
*floR_2*	Y	N	N	N	N	N	N	N	Y	N	Y	N	N	Y	N
*fosA_14*	N	N	N	N	N	N	N	N	Y	N	N	N	N	N	N
*glmM*	Y	N	Y	N	N	N	N	N	N	N	N	N	N	N	N
*mcr-1*	N	N	N	N	N	N	N	N	N	N	Y	N	N	N	N
*mph*(A)	Y	Y	N	N	N	Y	N	Y	N	Y	Y	Y	Y	Y	Y
*mphR*	Y	Y	N	N	N	Y	N	Y	N	Y	Y	Y	Y	Y	Y
*mrx*	Y	Y	N	N	N	Y	N	Y	N	Y	Y	Y	Y	Y	Y
*nimC*/*nimA*	N	N	N	N	N	N	N	N	N	N	Y	N	N	N	N
*oqxA*	Y	N	N	N	N	N	N	N	N	N	Y	N	N	N	N
*oqxB*	Y	N	N	N	N	N	N	N	N	N	Y	N	N	N	N
*oqxR*	Y	N	N	N	N	N	N	N	N	N	Y	N	N	N	N
*psp*	Y	N	N	N	N	N	N	N	N	N	N	N	N	N	N
*qacEd1*	Y	Y	N	N	N	Y	N	N	Y	Y	Y	Y	Y	Y	Y
*qacI*	Y	N	N	N	N	N	N	N	N	N	N	N	N	N	N
*qnrS1*	N	Y	N	N	N	N	N	Y	Y	N	N	N	N	N	N
*rmtB*	N	Y	N	N	N	N	N	N	Y	N	N	N	N	N	Y
*strA*	Y	Y	Y	N	N	N	N	Y	Y	N	Y	N	N	Y	N
*strB*	Y	Y	Y	N	N	N	N	Y	Y	N	Y	N	N	Y	N
*sul1*	Y	Y	N	N	N	Y	N	N	Y	Y	Y	Y	Y	Y	Y
*sul2*	Y	Y	Y	N	N	N	N	Y	Y	N	Y	N	N	Y	N
*sul3*	Y	N	N	N	N	N	N	N	N	N	N	N	N	N	N
*tet*(A)	Y	Y	Y	Y	N	Y	N	Y	Y	Y	Y	N	N	Y	Y
*tet*(B)	N	N	N	N	N	N	N	N	N	N	N	Y	Y	N	N
*tetC*	N	N	N	N	N	N	N	N	N	N	N	Y	Y	N	N
*tetD*	N	N	N	N	N	N	N	N	N	N	N	Y	Y	N	N
*tet*(M)	Y	N	N	N	N	N	N	N	N	N	N	N	N	N	N
*tetR*	Y	Y	Y	Y	N	Y	N	Y	Y	Y	Y	N	N	Y	Y
*tetR*(B)	N	N	N	N	N	N	N	N	N	N	N	Y	Y	N	N
*tmrB*	N	N	Y	Y	N	N	N	N	N	N	Y	Y	Y	N	N

a Y, yes (present); N, no (not present).

b AR genes, antibiotic resistance genes, including resistance genes carried on both plasmids and chromosomes and their associated transcriptional regulators. Multiple copies of the same genes were counted only once each. Numbers in parentheses represent all copies of genes.

c No. of AR genes near IS*26*, number of antibiotic resistance genes, including multiple copies of same gene, in a cluster within 20 kb of an IS*26* element. Antibiotic genes found within 10 kb of each other were considered to be part of the same gene cluster.

d A gene with a GNAT domain and overlapping with a transposase.

### Comparative genome sequence analysis of the Sanji chromosome.

**FIG 3:**
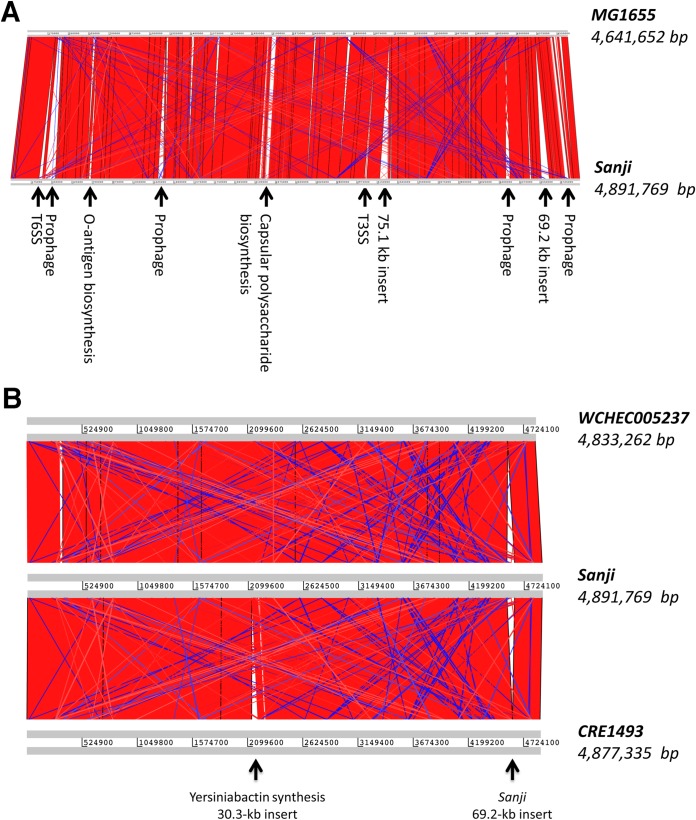
Synteny between the genomes of Sanji and related E. coli strains. (A) A pairwise genome comparison plot showing collinearity of genes between Sanji and MG1655. The 10 major insertions in the Sanji genome are labeled. The location of the capsular polysaccharide biosynthesis gene cluster in the Sanji corresponds to that of a lipopolysaccharide biosynthesis gene cluster in MG1655. All other insertions in MG1655 appear to be prophage-related genes. (B) A synteny plot comparing Sanji with two of the ST167 strains, CRE1493 and WCHEC005237. The 69.2-kb insertion is present only in Sanji, while the 30.3-kb insertion conferring yersiniabactin biosynthesis is present only in CRE1493. Each of the strains also has a few unique prophage insertions. The red and blue bands represent the forward and reverse matches, respectively.

At the time of Sanji genome completion, the closest genome available was that of prototypic E. coli K-12 strain MG1655. Genome alignment of Sanji chromosome to MG1655 revealed that 77% of the open reading frames in Sanji are shared with MG1655. A synteny plot generated based on the genome alignment between Sanji and MG1655 showed high collinearity with 10 major insertions (Fig. 3A). Since then, many additional genomes within the K-12 clade showing close relationships with Sanji have become available. Comparison of Sanji with two closely related strains, WCHEC005237 and CRE1493, revealed even greater collinearity (Fig. 3B).

**FIG 4:**
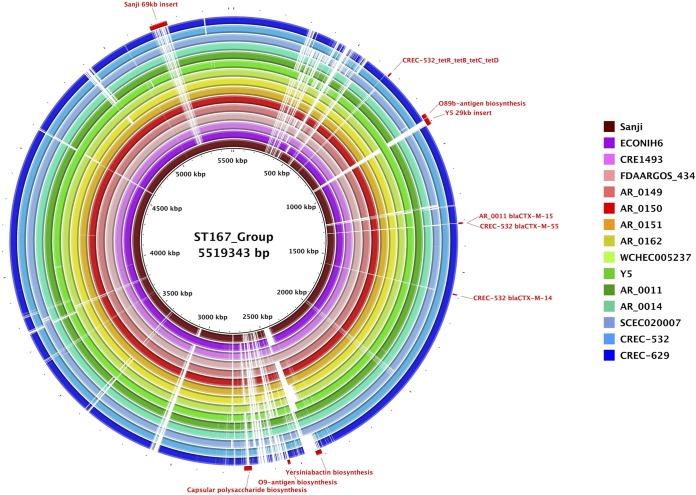
Comparisons of ST167 chromosomes. Shown is a BRIG circular genome plot for BLASTN comparisons of Sanji and 14 other ST167 strains. The reference sequence was a composite generated by inserting DNA segments into the Sanji chromosome that were absent from Sanji. Selected gene clusters involved in antibiotic resistance, O-antigen biosynthesis, capsular polysaccharide biosynthesis, and virulence are labeled.

Multilocus sequence typing (MLST) analysis using seven housekeeping genes (*purA*, *adk*, *icd*, *fumC*, *recA*, *mdh*, and *gyrB*) (28) classified Sanji into the sequence type ST167 group. Genome BLAST searches, using the unique insertions identified in comparisons with MG1655 as the query, revealed additional genomes that share some of these unique features, including strains with sequence types ST10, ST167, and ST617. An MLST-based phylogenetic tree of these strains revealed that these sequence types are indeed related to each other and fall within the K-12 clade (Fig. S1). Comparative genome sequence analysis of the entire chromosome of Sanji with the other 14 ST167 strains (Fig. 4) further revealed that the ST167 genomes are highly similar beyond the seven genes used for MLST. Some of these strains contain up to 12 distinct resistance genes on the chromosome (see Table 1).

10.1128/mSystems.00242-18.1 FIG S1 Phylogenetic analysis based on the seven genes used for MLST. A phylogenetic tree was constructed by the maximum likelihood method in the MEGA7 program using seven concatenated housekeeping genes of selected Escherichia coli strains, including those identified by BLAST searches using unique Sanji features as queries. The strains harboring the Sanji O89b biosynthesis gene cluster are identified with the following symbols: green circles, ST10 strains; red circles, ST167 strains; blue circles, ST617 strains; magenta squares, strains of other sequence types. The Sanji strain (highlighted in yellow) clustered with 14 other ST167 strains. Download FIG S1, PDF file, 0.1 MB. Copyright © 2019 Zeng et al. 2019 Zeng et al. This content is distributed under the terms of the Creative Commons Attribution 4.0 International license.

In comparison to MG1655, four of the chromosomal insertions in Sanji appear to be prophages (see Fig. 3A). Three insertions also found in other ST167 strains harbor specialized secretion systems (SS), namely, a 19.8-kb insertion containing a type 3 secretion system (T3SS), a 30.6-kb insertion containing a T6SS, and a 75.1-kb insertion containing a T2SS, although in some strains this insertion is truncated. Each of these insertions contains additional uncharacterized genes.

A 17.5-kb insertion containing an O-antigen biosynthesis cluster, flanked by a pair of insertion sequence 26 (IS*26*) elements, is shared with other ST167 strains, suggesting horizontal acquisition. Initial immunoserotyping analysis of the O-antigen gave positive results for type O6 but was unable to determine the H-type. PCR analysis failed to confirm the O6 serotype but gave positive results for H9 antigen. *In silico* serotyping based on the whole-genome sequence assigned the Sanji strain as serotype H9 based on the presence of the *fliC* gene sequence (98.9%). For the O-antigen, the closest match was related to serotype O89, based on the presence of *wzm* (94.1%) and *wzt* (93.5%). This newly determined 17.5-kb O-antigen gene cluster (≥99% sequence identity) was found to be present in all ST167 and ST617 strains examined, as well as in some strains within the ST10 clonal complex, including ST744, ST44, ST4981, ST1284, and ST10 (Fig. S1) (Table S1). We propose to designate this *in silico* serotype “O89b.” A few of the O89b-containing strains have additional genes encoding other O-antigen types, including O9 (based on genes *wzm* and *wzt*) or O8 (based on a truncated *wzt* gene). With the exception of a few strains, all of the ST167, ST617, and ST10 complex strains examined are predominantly H9 or H10 (Table S1).

10.1128/mSystems.00242-18.8 TABLE S1 *In silico* MLST analysis and serotyping of the E. coli Sanji genome and related E. coli genomes. Download Table S1, PDF file, 0.1 MB. Copyright © 2019 Zeng et al. 2019 Zeng et al. This content is distributed under the terms of the Creative Commons Attribution 4.0 International license.

**FIG 5:**
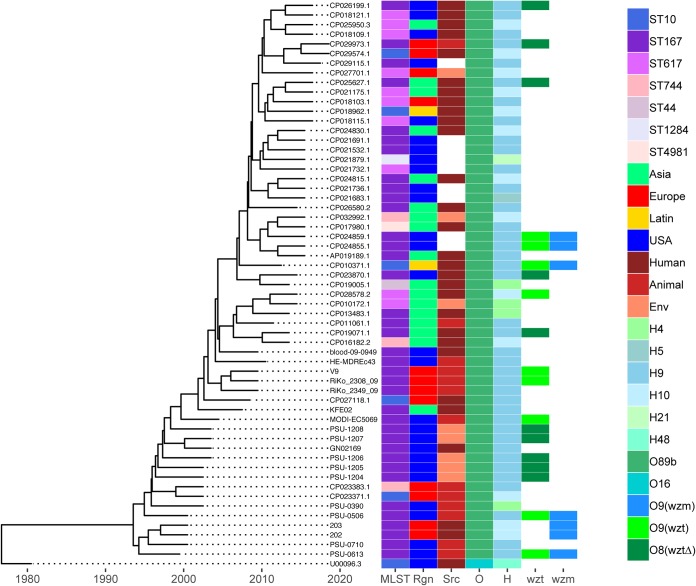
Time-scaled phylogeny of O89b-containing E. coli strains. Molecular phylogeny based on the HKY85 model was calculated using a BEAST2 MultiTypeTree module of 6,890 core SNVs from 39 Sanji-related complete genomes plus 19 ST167 assemblies isolated between 1999 and 2010, as well as MG1655. The tree shown was generated by using R package ggtree, with associated 7-gene MLST, isolation region, source, and *in silico* O-antigen and H-antigen serotypes indicated.

Maximum likelihood phylogenetic analysis of these O89b-containing strains was performed using MEGA7 for 6,890 core single nucleotide variants (SNV) across 39 Sanji-related genomes plus 19 ST167 assemblies and MG1655 (Fig. S2). Here, Sanji clustered with the early isolates of ST167, while later ST167 isolates showed more diversity. The ST617 isolates examined were less tightly clustered. The ST744, ST44, and ST10 isolates were more distant than the ST167 and ST617 groups. Using the same core SNV data set, the molecular evolution of these O89b-containing strains was also determined by a time-scaled Bayesian phylogenetic analysis in BEAST2 (29). From this analysis, it was estimated that development of these O89b-containing lineages took place about 30 years ago (Fig. 5). However, there is no clear geographical location associated with this emergence since members of this group appear to be dispersed globally. There also has been no clear time-dependent shifting of these lineages, though it appears that ST167 and ST617 are the dominant O89b-containing strains. ST617 strains are also known to carry many antibiotic resistance genes (30–33).

10.1128/mSystems.00242-18.2 FIG S2 Phylogenetic analysis based on 6,890 core SNVs of O89b-containing genomes and assemblies. A phylogenetic tree was constructed by the maximum likelihood method in the MEGA7 program using 6,890 core SNVs of the O89b-containing E. coli strains, plus MG1655. Sequence types are indicated. Download FIG S2, PDF file, 0.02 MB. Copyright © 2019 Zeng et al. 2019 Zeng et al. This content is distributed under the terms of the Creative Commons Attribution 4.0 International license.

A 32.7-kb insertion in Sanji contains a capsular polysaccharide biosynthesis (*cps*) gene cluster at a location that corresponds to a lipopolysaccharide biosynthesis gene cluster in MG1655. This *cps* gene cluster, flanked by IS elements, is also present in E. coli strains 127 and WCHEC005237 and has sequence homology with several K30 Klebsiella pneumoniae strains (28) but is truncated in several other ST167 strains (see Fig. 4).

A 69.2-kb insertion, unique to Sanji among the ST167 strains, contains the 8.9-kb antibiotic resistance gene cluster (see Fig. 2A), a raffinose utilization operon (*rafRABDY*), two toxin-antitoxin systems (*relE*/*parE* and *yeeV*/*yeeU*), and a number of unidentified genes. This insertion was also found in the chromosome of six other non-ST167 E. coli genomes (strains HB-Coli0, CRE1540, H8, MRY15-117, 14EC017, and WCHEC4533) (Table S2).

10.1128/mSystems.00242-18.9 TABLE S2 E. coli genomes carrying the 69.2-kb insertion. Download Table S2, PDF file, 0.1 MB. Copyright © 2019 Zeng et al. 2019 Zeng et al. This content is distributed under the terms of the Creative Commons Attribution 4.0 International license.

In addition to these major insertions, there are smaller insertions containing metabolic and nutrient acquisition genes, such as a 5.5-kb sucrose utilization operon (*cscBKAR*) shared with other ST167 strains. There were no other obvious toxins or other unique virulence factors that distinguished Sanji from the other ST167 strains. However, Sanji did exhibit *in vitro* growth inhibition against a laboratory strain of E. coli TOP10 expressing green fluorescence protein (GFP) (Fig. S3).

10.1128/mSystems.00242-18.3 FIG S3 Growth inhibition assay of E. coli Sanji compared to E. coli TOP10. Overnight cultures of E. coli TOP10 and E. coli Sanji were adjusted to an optical density at 600 nm (OD_600_) of 10 with fresh LB broth. Cultures (5 μl) of TOP10 alone were spotted onto an agar plate (A), and cultures of a mixture of TOP10 and Sanji were spotted onto separate agar plates at a ratio of 100:1 (B), 10:1 (C), or 1:1 (D). After incubation at 37°C for 2 hours, the agar discs containing the cells were excised and resuspended in 2 ml of LB broth. The resuspended cells were diluted 10^4^-fold with LB broth, and 100 μl of this dilution was plated and incubated at 37°C overnight and then at room temperature afterward. The green colonies were visible after 3 days. The image shown was taken 12 days after plating. Download FIG S3, PDF file, 1.6 MB. Copyright © 2019 Zeng et al. 2019 Zeng et al. This content is distributed under the terms of the Creative Commons Attribution 4.0 International license.

### Comparative sequence analysis of the Sanji plasmids.

**FIG 6:**
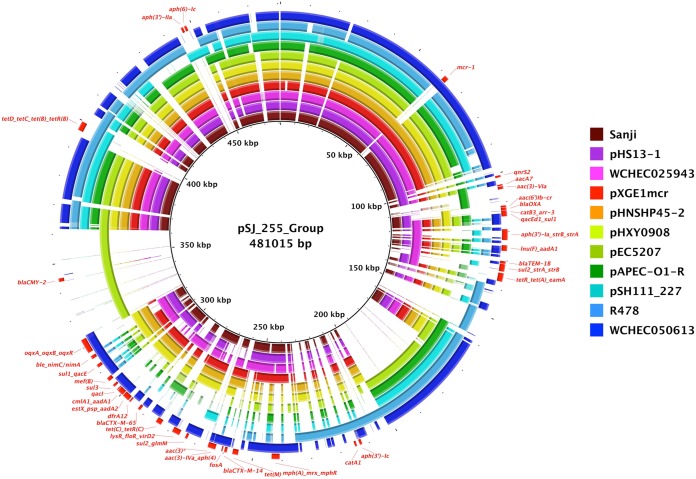
Comparisons of plasmids related to pSJ_255. Shown is a BRIG circular plot for BLASTN comparisons of pSJ_255 and related plasmids. The reference sequence is a composite generated by inserting sequences into the pSJ_255 sequence that were absent from pSJ_255. Each ring corresponds to a different plasmid, as follows from inner to outer ring: pSJ_255 represents plasmid pSJ_255 from E. coli Sanji; pHS13-1 represents plasmid pHS13-1 from E. coli HS13-1; WCHEC025943 represents plasmid pMCR1_025943 from E. coli WCHEC025943; pXGE1mcr represents plasmid pXGE1mcr from E. coli XG-E1; pHNSHP45-2 represents plasmid pHNSHP45-2 from E. coli SHP45; pHXY0908 represents plasmid pHXY0908 from Salmonella enterica serovar Typhimurium strain GDS147; pEC5207 represents plasmid pEC5207 from E. coli EC5207; pAPEC-O1-R represents plasmid pAPEC-O1-R from E. coli APEC O1; pSH111_227 represents plasmid pSH111_227 from *Salmonella* Heidelberg; R478 represents plasmid R478 from Serratia marcescens; and WCHEC050613 represents plasmid pMCR_WCHEC050613 from E. coli WCHEC050613. All identifiable antibiotic resistance genes are labeled in red on the outer ring.

For most ST167 genomes, including Sanji, the majority of their antibiotic resistance genes were located on various plasmids (Table 1). Interestingly, all of the strains carried distinct sets of plasmids with different backbones and sizes (Table 2). The IncHI2 plasmid, pSJ_255, is unique to Sanji among ST167 strains and carries 27 of the 32 distinct antibiotic resistance genes found in Sanji (Fig. 2C). This plasmid belongs to a family of plasmids whose prototypical member is Serratia marcescens plasmid R478 (34) (Fig. 6). This family of plasmids contains the *ter* gene cluster, which has been shown to confer resistance to tellurite, some bacteriophages, and pore-forming colicins (35, 36). MDR plasmids in this family differ in the number of antibiotic resistance genes (Table S3). For example, R478 and a few others carry 4 to 8 antibiotic resistance genes, while others, including pSJ_255, carry 23 to 30. Additionally, each of these plasmids carries a different but overlapping set of antibiotic resistance genes.

**TABLE 2:** Plasmid MLST types associated with ST167 E. coli strains

Parameter	Value(s) for E. coli strain:														
	Sanji	ECONIH6	AR_0011	AR_0014	AR_0149	AR_0150	AR_0151	AR_0162	WCHEC005237	FDAARGOS_434	CRE1493	CREC-532	CREC-629	Y5	SCEC020007
No. of plasmids (PubMLST)	6	2	3	2	2	3	2	4	8	1	5	3	3	3	2

Plasmid size (bp)^*a*^															
FIA_4; FIA_20; FII_36	94,712 (0)					117,703 (10)				149,485 (24)	127,772 (15)			124,378 (13)	144,225 (24)
FIA_1; FIA_6; FII_22; FII_36												216,181 (17)	176,274 (15)		
FIA_4; FIA_20; FIB_1; FII_31; FII_36			181,436 (8)	172,588 (8)											
FIB_24											73,992 (18)				
FII_2	82,288 (0)	100,989 (12)						84,929 (5)							
FII_33									70,691 (2)						
FII_47									100,229 (12)						
HCM1 178ac_2 IncHI1				33,548 (0)											
IncHI2 DLST ST 3	255,368 (27)														
smr0018 IncHI2	2,640 (0)														
ardA_4; repI1_1; trbA_15; IncI1														61,695 (2)	
ardA_4; repI1_3; trbA_15; IncI1						50,235 (2)	50,228 (2)								
ardA_5; repI1_4; trbA_15; IncI1					48,528 (1)										
ardA_19 IncI1															84,952 (0)
ardA_24 IncI1									6,200 (3)						
trbA_43 IncI1			57,991 (0)												
repN_6 IncN			76,680 (4)												
IncA/C ST 3														136,243 (8)	
A009_11 IncA/C								23,332 (0)			33,858 (1)				
A157: 6 IncA/C		97,800 (10)						95,850 (8)							
A165_4 IncA/C	98,436 (1)										96,986 (0)	96,987 (0)	96,990 (0)		
A165_6 IncA/C	3,373 (0)								3,684 (0)						
A175_5 IncA/C						46,159 (2)	46,161 (2)	49828 (2)	46,161 (2)		46145 (2)				
parB_9 IncA/C					46,161 (2)							46137 (2)	46,161 (2)		
repA_4 IncA/C									121,908 (9)						
None^*b*^									2,959 (0)						
None									2,444 (0)						
No. of chromosome-borne resistance genes	6	0	1	0	0	0	0	0	0	0	0	6	6	20^*c*^	0

a Numbers indicate plasmid sizes (bp). Numbers of resistance genes, including replicates and associated transcriptional regulators, are shown in parentheses.

b None, no match in PubMLST database.

c Value includes 3 copies of a 4-gene cluster.

10.1128/mSystems.00242-18.10 TABLE S3 Antibiotic resistance gene profiles of pSJ_255-related IncHI2 plasmids. Download Table S3, PDF file, 0.1 MB. Copyright © 2019 Zeng et al. 2019 Zeng et al. This content is distributed under the terms of the Creative Commons Attribution 4.0 International license.

One explanation for this high variability in the number and types of antibiotic resistance genes in SJ_255 is the presence of several IS*26* elements (Fig. 2C). IS*26* elements are known to facilitate the horizontal movement and accumulation of antibiotic resistance genes at a relatively high frequency (37, 38). Although pSJ_255 does not carry genes with resistance to the current “antibiotics of last resort” (e.g., *blaNDM* or *blaKPC*, conferring resistance to carbapenems, or *mcr-1*, conferring hyperresistance to colistin), several plasmids in the IncHI2 family have acquired the *mcr-1* gene (Fig. 6; see also Table S3). Moreover, a recent report identified an IncHI2 plasmid that carries both *blaNDM-4* and *mcr-1* (39).

Sanji plasmid pSJ_82 belongs to the IncFII family of plasmids, which includes prototypical member pHK01 (40). Members of this family carry the ESBL-encoding *blaCTX-M-14* gene (41). Sanji, ECONIH6, and AR_0162 all have a plasmid with an FII_2 backbone (SJ_82, tig00008015, and pNDM-d2e9, respectively), carrying 0, 12, and 5 distinct antibiotic resistance genes, respectively (Fig. S4). Sanji plasmid pSJ_94 carries both IncFIA and IncFII replicons but no identifiable antibiotic resistance genes (Fig. S5). However, it does contain an IS*26* element. In fact, close relatives of both pSJ_94 and pSJ_82 carry IS*26* elements and a large number of associated antibiotic resistance genes (see Fig. S4 and S5).

10.1128/mSystems.00242-18.4 FIG S4 BRIG circular gene plot comparison of plasmids related to pSJ_82. The reference sequence is a composite generated by inserting sequences present in other plasmids but absent in pSJ_82 into the pSJ_82 sequence. Each ring corresponds to a different plasmid as follows from inner to outer ring: pSJ_82 represents plasmid pSJ_82 from E. coli Sanji; pKP04CTXM from Klebsiella pneumoniae *KP04*; pHK01 from E. coli Combat2D2; RCS62_pI from E. coli *506*; pKF3-70 from Klebsiella pneumoniae *KF3*; pEC13 from an unknown E. coli strain; tig00008015 from E. coli *AR_0162*; and pNDM-d2e9 from E. coli *ECONIH6*. All identifiable antibiotic resistance genes are labeled in red on the outer ring. Download FIG S4, PDF file, 0.3 MB. Copyright © 2019 Zeng et al. 2019 Zeng et al. This content is distributed under the terms of the Creative Commons Attribution 4.0 International license.

10.1128/mSystems.00242-18.5 FIG S5 BRIG circular gene plot comparison of plasmids related to pSJ_94. The reference sequence is a composite generated by inserting sequences present in other plasmids but absent in pSJ_94 into the pSJ_94 sequence. Each ring corresponds to a different plasmid as follows from inner to outer ring: pSJ_94 represents plasmid pSJ_94 from E. coli Sanji; AR_0011 represents plasmid tig00001011_pilon from E. coli AR_0011; AR_0014 represents plasmid unitig_1_pilon from E. coli AR_0014; pCREC-532_1 represents plasmid pCREC-532_1 from E. coli CREC-532; pCREC-629_1 represents plasmid pCREC-629_1 from E. coli CREC-629; AR_0150 represents plasmid tig00000255 from E. coli AR_051; p1493-5 represents plasmid p1493-5 from E. coli CRE1493; pECY55 represents plasmid from E. coli Y5; FDAARGOS_434 represents plasmid unnamed1 from E. coli FDAARGOS_434; and SCEC020007 represents plasmid pNDM5_0200007 from E. coli SCEC020007. All identifiable antibiotic resistance genes are labeled in red on the outer ring. Download FIG S5, PDF file, 0.4 MB. Copyright © 2019 Zeng et al. 2019 Zeng et al. This content is distributed under the terms of the Creative Commons Attribution 4.0 International license.

Among the ST167 strains analyzed, the majority of their antibiotic resistance genes are associated with IS*26* elements (see Table 1). Sanji pSJ_255 carries 24 IS*26*-associated antibiotic resistance genes. ST167 strain FDAARGOS_434 carries a plasmid related to pSJ_94 with 24 IS*26*-associated resistance genes, and strain ECONIH6 carries a plasmid related to pSJ_82 that has 12 IS*26*-associated resistance genes. In Sanji, the ESBL encoded by *blaCTX-M-14* appears to be mobilized by ISEcp1 and is not associated with IS*26* elements. However, there has been a report of a *blaCTX-M-14_ISEcp1* gene cluster that inserted into an IS*26* element in a strain of *Proteus* (42), while there have been multiple reports of the *blaCTX-M-15* gene being associated with IS*26* elements in E. coli (43, 44). Several of the ST167 strain plasmids also contained Tn*3*-mediated insertions of the *blaCTX-M-15* gene into IS*26* elements (Fig. S5). The existence of these related plasmids containing IS*26* elements suggests that pSJ_94 and pSJ_82 in Sanji have the potential to also accumulate multiple antibiotic resistance genes in a manner similar to that observed for pSJ_255.

Sanji plasmid pSJ_98 appears to be a P1-like enterobacteriophage. Closely related plasmids can be found in many bacteria, including some ST167 strains, CRE1493, CREC-532, and CREC-629 (Fig. S7). In rare cases, these plasmids can carry an antibiotic resistance gene, but there is no evidence for accumulation of multiple resistance gene clusters such as was observed with the other large Sanji plasmids.

10.1128/mSystems.00242-18.6 FIG S6 Operon structure of Tn*3*-mediated insertions of *blaCTX-M-15* into IS26 elements in ST167 strains. Plasmids p1493-5 from E. coli CRE1493, pECY55 from E. coli Y5, and unnamed1 from E. coli FDAARGOS_434 contain a *blaCTX-M-15-Tn3* gene cluster flanked by IS*26* elements, while plasmids unitig_1_pilon from E. coli AR_0014 and tig00001011_pilon from E. coli AR_0011 have an *ISEcp1* gene as well. The *blaCTX-M-15* genes are indicated in cyan; IS*26* elements in magenta; and Tn*3* transposase genes in green. Download FIG S6, PDF file, 0.1 MB. Copyright © 2019 Zeng et al. 2019 Zeng et al. This content is distributed under the terms of the Creative Commons Attribution 4.0 International license.

10.1128/mSystems.00242-18.7 FIG S7 BRIG circular gene plot comparison of plasmids related to pSJ_98. The reference sequence is a composite generated by inserting sequences present in other plasmids but absent in pSJ_98 into the pSJ_98 sequence. Each ring corresponds to a different plasmid as follows from inner to outer ring: pSJ_98 represents plasmid pSJ_98 from E. coli Sanji; p1493-4 represents plasmid p1493-4 from E. coli CRE1493; pCREC-629_2 represents plasmid pCREC-629_2 from E. coli CREC-629; pBJ114-96 represents plasmid pBJ114-96 from E. coli BJ114; p1303_95 represents plasmid p1303_95 from E. coli 1303; pMS6198C represents plasmid pMS6198C from E. coli *MS6198*; 127_p91 represents plasmid p91 from E. coli *127*; and U2501 represents plasmid U2501 from E. coli WE-0250. The *blaCTX-M-15* gene is the only antibiotic resistance gene observed in one member of this plasmid family and is labeled in red. Download FIG S7, PDF file, 0.9 MB. Copyright © 2019 Zeng et al. 2019 Zeng et al. This content is distributed under the terms of the Creative Commons Attribution 4.0 International license.

### Multiple paths to achieve XDR.

ESBL-producing MDR and XDR E. coli isolates with sequence types ST167 and ST617 and others in the ST10 clonal complex have emerged over the past 5 years as common isolates from nosocomial sources as well as wild animal and domestic animal sources, including dairy and livestock sources, in Germany (45–47), Taiwan (48, 49), Tunisia (50, 51), and the Americas (52). Indeed, ST167 strains carrying carbapenemase activity have become the second most prevalent sequence type, behind only ExPEC ST131, among human clinical ESBL-producing E. coli isolates reported in China (27, 53–62), Spain (63, 64), France (65), India (66), Italy (67), Iran (68), Romania (69), and Tunisia (50).

Because IS*26* elements can be readily exchanged between different DNA molecules (transposons, phages, conjugated plasmids, transformed DNA chromosomes, etc.), bacteria that can acquire multiple IS*26*-containing plasmids can facilitate the generation of expanded gene clusters with multiple antibiotic resistance genes. This process is accelerated under conditions conducive to the coalescence of diverse bacterial strains that are also amenable to horizontal gene transfer. The animal gut has been shown to be particularly conducive to high rates of conjugal transfer between bacteria under conditions of inflammation or disease (70–72). These disease conditions frequently coincide with administration of antibiotics, creating a strong selective pressure for accumulation of genes that confer antibiotic resistance.

The observed diversity in the number and type of antibiotic resistance genes and the diverse mechanisms for their spreading among Sanji and related O89b-containing E. coli strains indicate that acquisition of XDR properties can occur through multiple evolutionary paths. One implication of this observation is that targeted elimination of any existing XDR strain is unlikely to prevent the emergence of new strains with similar XDR properties. A second implication is that even the best antibiotic stewardship is unlikely to be sufficient to prevent or mitigate the development of XDR pathogens. These potential consequences underscore the urgency of the quest for better understanding the shared physiology and virulence mechanisms of pathogenic bacteria, such as the group identified here with O89b-antigens.

## MATERIALS AND METHODS

### Isolation and serotyping of E. coli strain Sanji.

E. coli strain Sanji was isolated from the duodenum of a pheasant during a 2011 outbreak of fowl colibacillosis on a farm in Fujian province, China, that had about 400 pheasants. Symptoms included drooping, anorexia, diarrhea, soft feet, and inability to flutter or fly. The disease was refractory to common veterinary antibiotics, including amikacin, which was administered after drug sensitivity testing during the second week of the outbreak. Within 1 month, all of the pheasants became severely ill and died or had to be culled. Serotyping of E. coli Sanji, which formed mucoid colonies on LB agar plates, was performed by the Tianjin Biochip Corporation.

### Antibiotic susceptibility profiling of E. coli strain Sanji.

Antibiotic susceptibility testing was performed using the Kirby-Bauer disk diffusion method with test discs (6.5-mm to 7.0-mm diameter), according to Clinical & Laboratory Standards Institute (CLSI) M100 guidelines (https://clsi.org). Antibiotic susceptibility to colistin (Arcos) and polymyxin B (Sigma) was assayed using the broth microdilution method, according to EUCAST guidelines (www.eucast.org). Reference E. coli strain ATCC 25922 and Kirby-Bauer test discs were obtained from Hangzhou Tianhe Microorganism Reagent Co., and reference E. coli strain MG1655 was obtained from Miao Ling Bio (Wuhan, China).

### Genome sequencing, assembly, and annotation of the E. coli Sanji genome.

Total genomic DNA was prepared using a Qiagen Genomic-tip kit, according to the manufacturer's protocol. Illumina sequencing was performed at the Beijing Genomics Institute (BGI; Shenzhen, People’s Republic of China) using a HiSeq 2000 platform with insertions of 484 bp and 6,354 bp. Assembly of the 815 Mb of 90-bp read-length paired-end sequencing data generated from the Illumina platform was unable to close the genome, so we applied a PacBio SMRT sequencing and *de novo* assembly platform. For PacBio sequencing, library construction, sequencing, assembly, and annotation were performed by Pacific Biosciences (Menlo Park, CA), using a PacBio RS II system. Totals of 518,559,882 and 306,969,330 postfilter bases from the size-selected and non-size-selected libraries were obtained with mean subread lengths of 6,292 and 1,590, respectively. The size-selected library assay was performed using a BluePippin system (SageScience) to remove shorter DNA insertions with a size cutoff of ≤15 kb. The non-size-selected library was also included to capture and sequence the smaller 3.4-kb and 2.6-kb plasmids. A total of 839,222,725 bases were assembled using the HGAP (v. 2.3) long-read assembler (73) into 15 polished contigs (maximum contig length of 4,926,777) with mean coverage of 135×. The resulting genomes of the single circular chromosome (4,891,769 bp) and six circular plasmids (255,368 bp, 98,436 bp, 94,712 bp, 82,288 bp, 3,373 bp, and 2,640 bp) were annotated using the best-placed reference protein set (GeneMarkS+) in the NCBI Prokaryotic Genome Annotation Pipeline (ver. 3.3).

### *In silico* serotyping, antibiotic resistance gene profiling, and IS element analysis of E. coli strain Sanji.

Sequence-based bacterial serotyping was performed using SerotypeFinder (ver. 1.1) at https://cge.cbs.dtu.dk/services/SerotypeFinder/ (74). Antibiotic resistance genes were identified using blastn against a database generated from the Resfams database at www.dantaslab.org/resfams (75), the ResFinder database at www.genomicepidemiology.org (76), and information obtained from the review published previously by Roberts et al. (77). A shell script was used to extract the list of antibiotic resistance gene clusters from the blastn output. Insertion sequence (IS) elements were identified using ISfinder at http://www-is.biotoul.fr (78).

### Comparative genome sequence analysis.

Genome sequences of E. coli ST167 strains (ECONIH6 [GenBank accession no. CP026199.1], AR_0150 [GenBank accession no. CP021736.1], AR_0151 [GenBank accession no. CP021691.1], AR_0149 [GenBank accession no. CP021532.1], WCHEC005237 [GenBank accession no. CP026580.2], FDAARGOS_434 [GenBank accession no. CP023870.1], CRE1493 [GenBank accession no. CP019071.1], AR_0014 [GenBank accession no. CP024859.1], AR_0011 [GenBank accession no. CP024855.1], AR_0162 [GenBank accession no. CP021683.1], CREC-532 [GenBank accession no. CP024830.1], CREC-629 [GenBank accession no. CP024815.1], Y5 [GenBank accession no. CP013483.1], SCEC020007 [GenBank accession no. CP025627.1], 51008369SK1 [GenBank accession no. CP029973.1], AR435 [GenBank accession no. CP029115.1], M217 [GenBank accession no. AP019189.1), ST617 strains (AR_0114 [GenBank accession no. CP021732.1], MRSN346355 [GenBank accession no. CP018121.1], MRSN346638 [GenBank accession no. CP018115.1], MRSN346595 [GenBank accession no. CP018109.1], MRSN352231 [GenBank accession no. CP018103.1], 5CRE51 [GenBank accession no. CP021175.1], SCEC020023 [GenBank accession no. CP025950.3], H8 [GenBank accession no. CP010172.1], WCHEC005784 [GenBank accession no. CP028578.2], 675SK2 [GenBank accession no. CP027701.1), ST10 strains (1283 [GenBank accession no. CP023371.1], Ecol_422 [GenBank accession no. CP018962.1], 6409 [GenBank accession no. CP010371.1], DA33133 [GenBank accession no. CP029574.1], 26561 [GenBank accession no. CP027118.1), ST744 strains (1223 [GenBank accession no. CP023383.1], EC590 [GenBank accession no. CP016182.2], W5-6 [GenBank accession no. CP032992.1), and other strains (Ecol_AZ155 [GenBank accession no. CP019005.1], CH611_eco [GenBank accession no. CP017980.1], AR_0137 [GenBank accession no. CP021879.1], MG1655 [GenBank accession no. U00096.3), including their plasmids, were used for comparative genome sequence analysis. Multilocus sequence typing (MLST) of the Sanji strain and comparison with sequences of other related E. coli strains were based on the use of Achtman's seven housekeeping genes for E. coli (*purA*, *adk*, *icd*, *fumC*, *recA*, *mdh*, and *gyrB*) (28) and performed using a shell script based on the Enterobase database at https://enterobase.warwick.ac.uk/species/ecoli/download_7_gene. Molecular phylogenetic analysis was performed by the maximum likelihood method based on the Tamura-Nei model (79) using MEGA7 (80) with 1,000 bootstrap iterations. Synteny plots were generated using Artemis Comparison Tool (ACT) software (81) and blastn results based on genome alignments. Circular plots for genome comparison were produced using BLAST Ring Image Generator (BRIG) (82). Plasmids were analyzed and typed by plasmid multilocus sequence typing (pMLST) using the PubMLST database (http://pubmlst.org/plasmid) (83). IS*26*-associated antibiotic resistance genes were defined as resistance genes located within 10 kb of an IS*26*-like element. Gene graphics were generated with the aid of SnapGene Viewer software (GSL Biotech).

The 39 Sanji-related genomes plus 19 ST167 assemblies, from isolates obtained between 1999 and 2010, and MG1655 were used to generate core single nucleotide variants (SNVs). The sequences of the 19 ST167 assemblies were downloaded from the Enterobase database at https://enterobase.warwick.ac.uk and concatenated as a continuous fasta file. The recurring regions and unique regions of the 40 complete genome sequences were removed using a shell script. This method is based on genome-to-genome blastn at 99% coverage and 99% identity and subsequent removal of recurring and unique regions. The Sanji genome reference template was used as a query for blastn analysis against another genome. The resulting common regions shared by the two genomes were then joined (as a “stitched” sequence) and used for blastn analysis of another new genome sequence to generate a new stitched sequence until all 40 of the genomes, including Sanji, were compared. This entire process was then repeated 10 times. After 6 iterations, a convergent, consensus stitched sequence of 2,493,769 bp was obtained. This consensus stitched sequence was then subjected to blast analysis against each individual genome, followed by the use of a shell script to remove all sites with gapped or identical sequences to generate a string of ungapped 6,890 core SNVs. The consensus core stitched sequence was used similarly to generate a string of 6,890 core SNVs for each of the 19 assemblies. This alignment of SNVs was used for modeling mutation rate estimates and time-scaled phylogeny using MEGA7 and BEAST2.5.1 packages.

The MultiTypeTree module of BEAST2 was used with the following parameters: (i) tip dates were set as the sample isolation dates (or as the database submission date for cases where no isolation date was provided); (ii) tip locations were set as three geographic regions (Americas, Asia, and Europe); (iii) the gamma site model was selected with the HKY85 nucleotide substitution model (84); and (iv) a strict clock model was used with an initial mutation rate set at 10^−10^ mutations per site per year. For the priors, a uniform distribution was selected for clockRate.c with an initial value of 10^−10^ and an upper limit of 10^−7^; exponential distribution was selected for gammaShape.s with an initial value of 1; log normal was selected for kappa.s with an initial value of 2; 1/*X* distribution was selected for popSizes.t; exponential distribution was selected for rate Matrix.t; and equal population sizes and a symmetric migration rate matrix were assumed for the migration model. In trial runs sampling 10^6^ Markov chain Monte Carlo (MCMC) steps, we explored HKY85, TN93, and generalized time-reversible (GTR) nucleotide substitution models with various parameters. The TN93 and GTR models could not accommodate mutation rates lower than 0.001, and even with the clock rate accepted by the module, runs were often terminated prematurely. For those runs that were completed, the models gave results comparable to those obtained with the HKY85 model. Using the HKY85 model, five runs with 10^8^ MCMC steps were performed, with 10% discounted as representing burn-in and a tree logging frequency of 10^5^. Tree files were combined using LogCombiner in the BEAST2 package, followed by the use of TreeAnnotator to annotate the combined trees. The annotated output trees file was used to generate the phylogenetic tree with associated metadata, including 7-gene MLST, isolation region (United States, Latin America, Europe, Asia), source (human, animal, environmental), and *in silico* O-antigen and H-antigen serotypes, using the R package ggtree.

### Data availability.

The complete genome and plasmid sequences of E. coli strain Sanji have been deposited in the NCBI under accession numbers CP011061.1 (circular chromosome; 4,891,769 bp), CP011062.1 (pSJ_255; 255,368 bp), CP011063.1 (pSJ_98; 98,436 bp), CP011064.1 (pSJ_94; 94,712 bp), CP011065.1 (pSJ_82; 82,288 bp), CP011066.1 (pSJ_3; 3,373 bp), and CP011067.1 (pSJ_2; 2,640 bp).
