# Supplementary figures and images for: Comparative Genome Analysis of an Extensively Drug-Resistant Isolate of Avian Sequence Type 167 Escherichia coli Strain Sanji with Novel In Silico Serotype O89b:H9
Source: mSystems. 2019 Feb 26;4(1):e00242-18. doi: 10.1128/mSystems.00242-18 (PMC6392093; doi:10.1128/mSystems.00242-18)

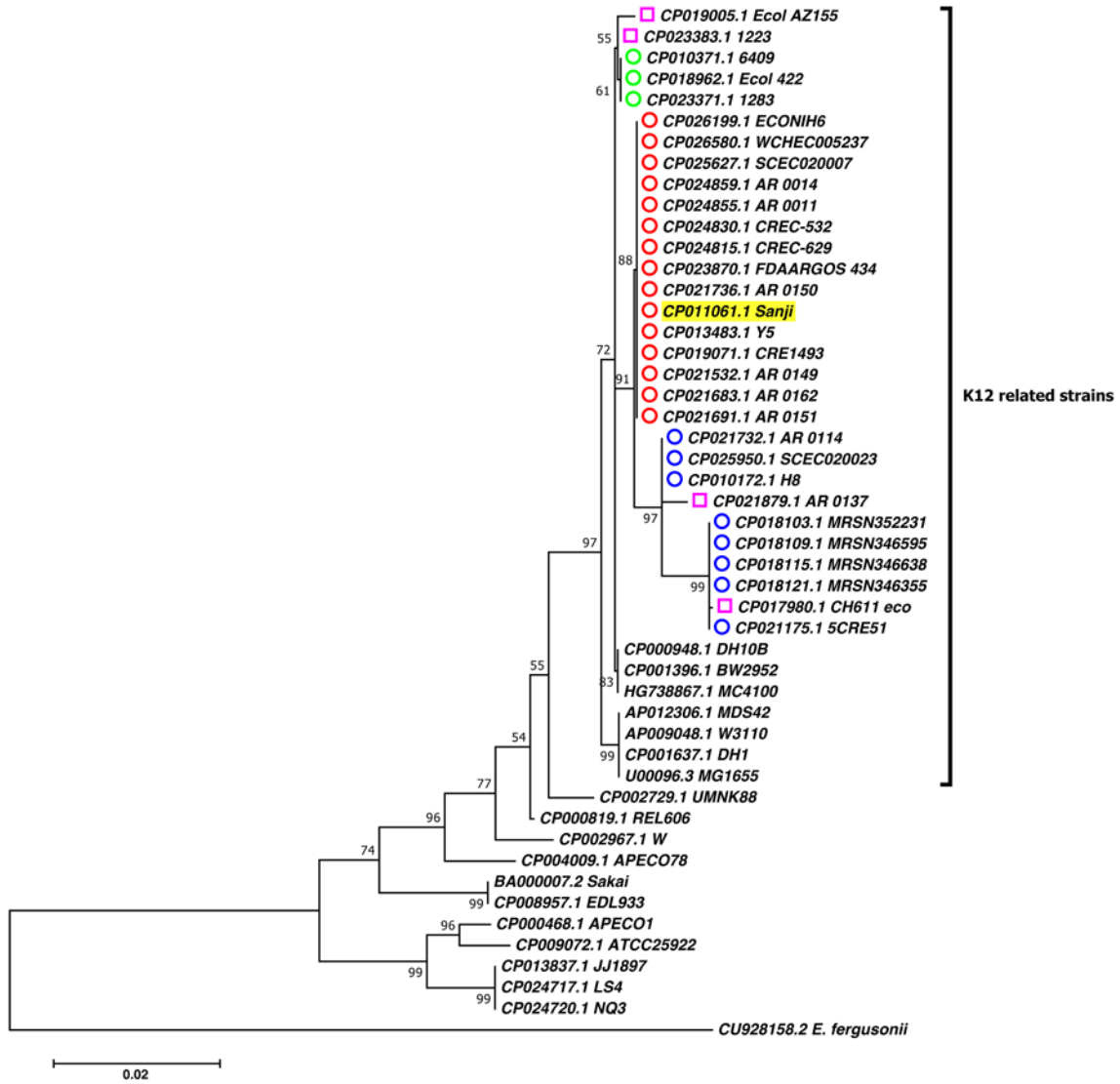

Supplement: FIG S1 [file mSystems.00242-18-sf001.pdf]

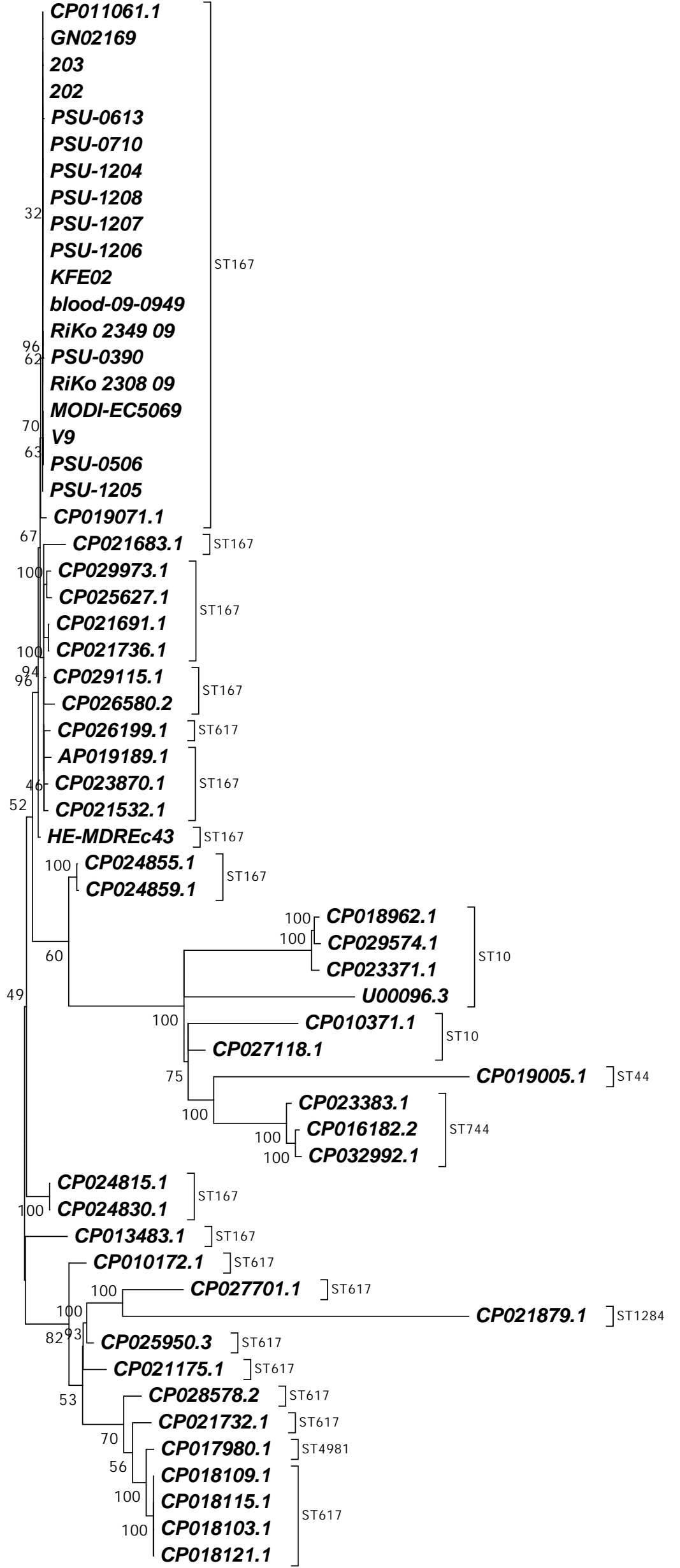

0.020

Supplement: FIG S2 [file mSystems.00242-18-sf002.pdf]

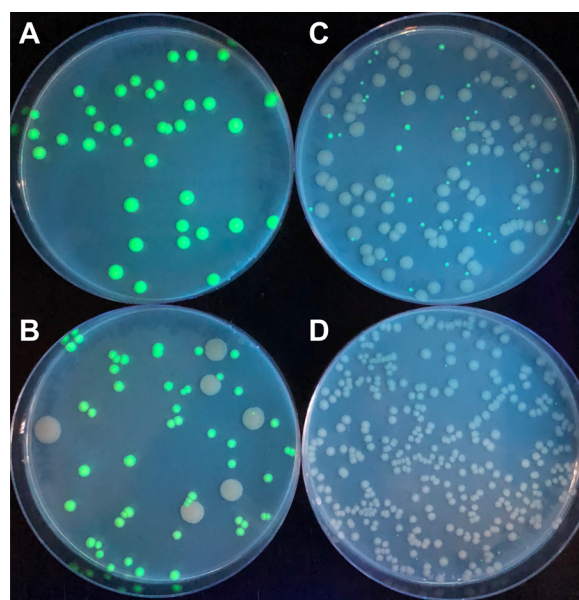

Supplement: FIG S3 [file mSystems.00242-18-sf003.pdf]

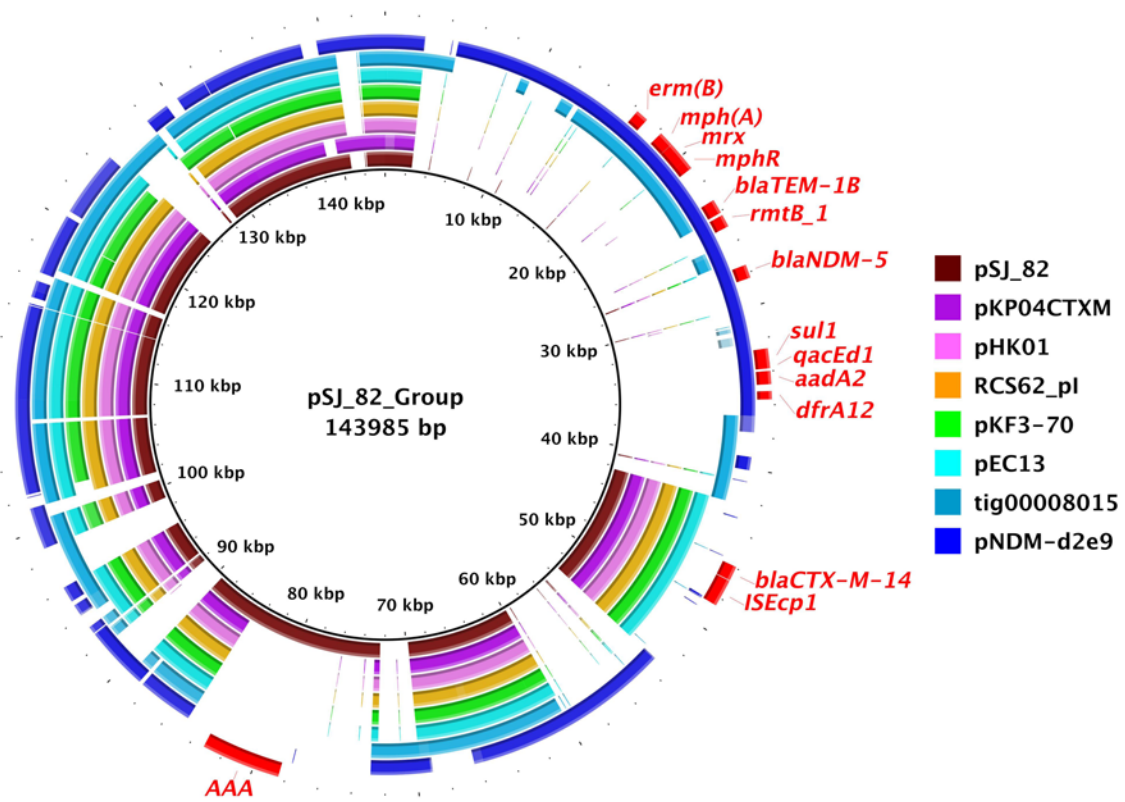

Supplement: FIG S4 [file mSystems.00242-18-sf004.pdf]

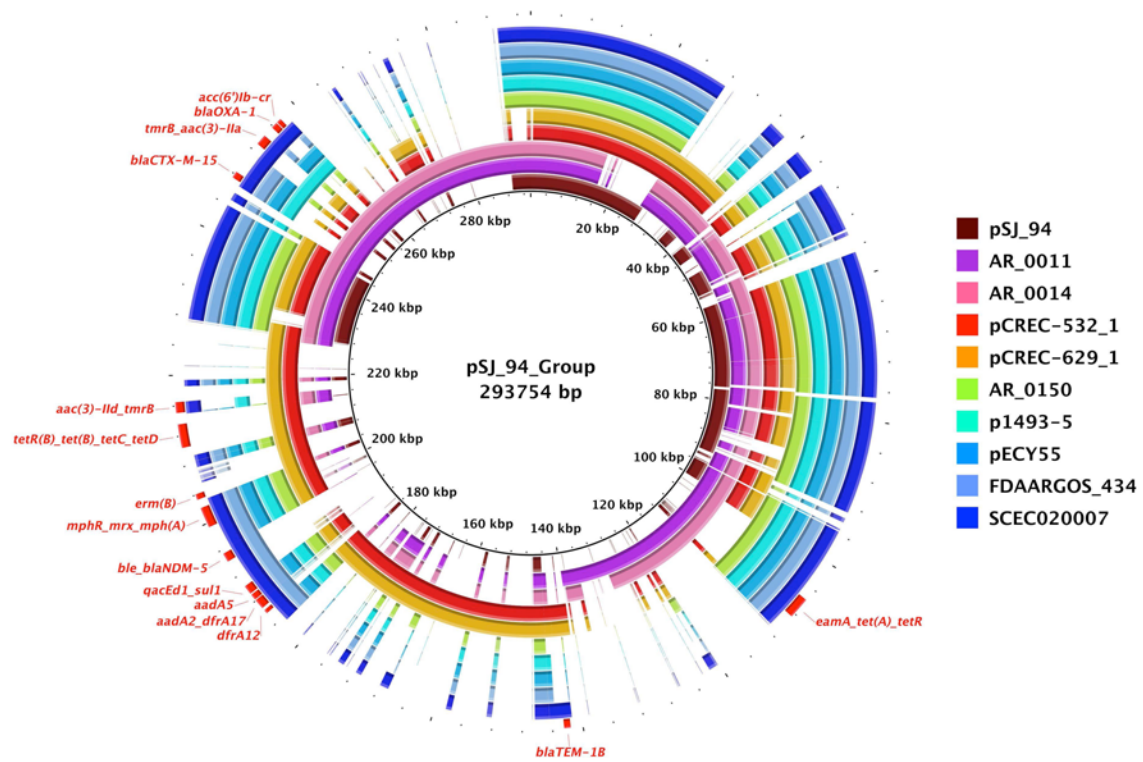

Supplement: FIG S5 [file mSystems.00242-18-sf005.pdf]

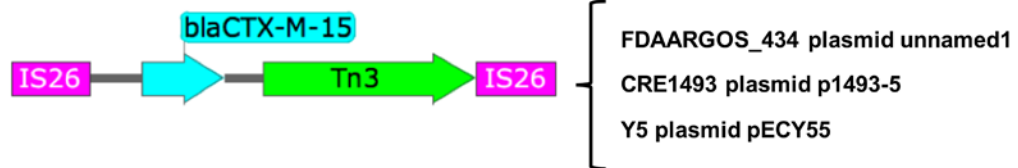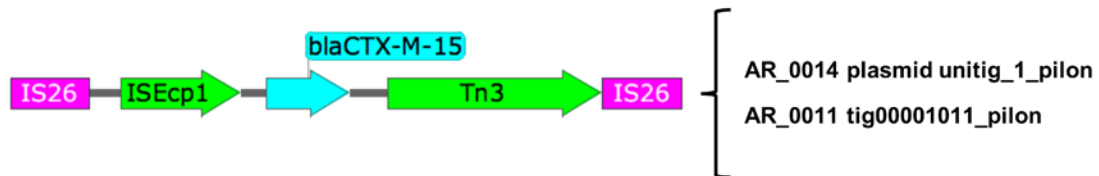

Supplement: FIG S6 [file mSystems.00242-18-sf006.pdf]

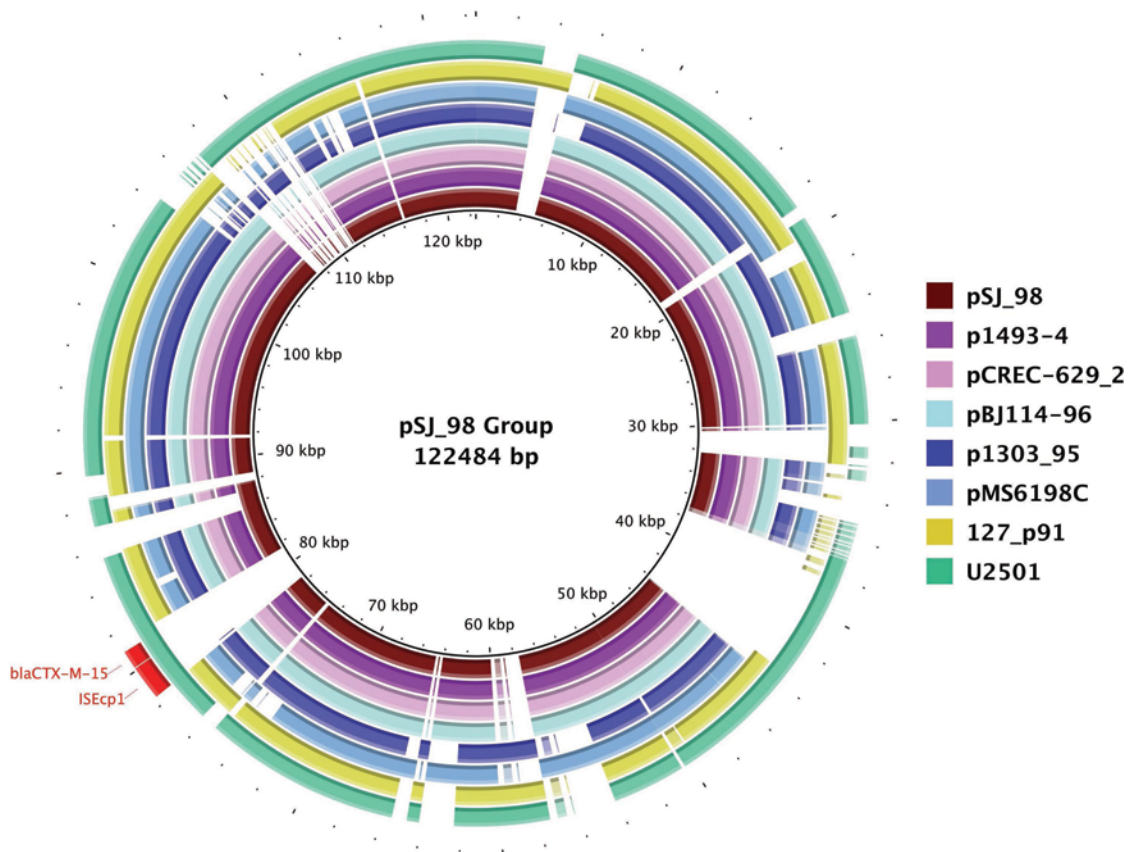

Supplement: FIG S7 [file mSystems.00242-18-sf007.pdf]
