# Supplementary material for: Comparative Genome Analysis of an Extensively Drug-Resistant Isolate of Avian Sequence Type 167 Escherichia coli Strain Sanji with Novel In Silico Serotype O89b:H9
Source: mSystems. 2019 Feb 26;4(1):e00242-18. doi: 10.1128/mSystems.00242-18 (PMC6392093; doi:10.1128/mSystems.00242-18)
Supplement: TABLE S1 [file mSystems.00242-18-st001.pdf]

Table S1. In silico MLST analysis and serotyping of *E. coli* Sanji genome and related *E. coli* genomes.

E. coli Strain	Accession No.	7-gene MLST	*fliC*	*wzm* (a)	*wzt* (a)	*wzm* (b)	*wzt* (b)	Homology (c)
**Sanji**	CP011061.1	ST167	H9	O89b	O89b			17499/17499
**CRE1493**	CP019071.1	ST167	H9	O89b	O89b		O8	17499/17499
**AR_0149**	CP021532.1	ST167	H9	O89b	O89b			17499/17499
**AR_0150**	CP021736.1	ST167	H9	O89b	O89b			17499/17499
**AR_0151**	CP021691.1	ST167	H9	O89b	O89b			17499/17499
**FDAARGOS_434**	CP023870.1	ST167	H9	O89b	O89b		O8	14833/17499 (d)
**SCEC020007**	CP025627.1	ST167	H9	O89b	O89b		O8	11852/17499 (d)
**ECONIH6**	CP026199.1	ST167	H9	O89b	O89b		O8	17499/17499
**WCHEC005237**	CP026580.2	ST167	H9	O89b	O89b			17496/17499
**AR_0011**	CP024855.1	ST167	H9	O89b	O89b	O9	O9	17499/18275 (e)
**AR_0014**	CP024859.1	ST167	H9	O89b	O89b	O9	O9	17499/17499
**51008369SK1**	CP029973.1	ST167	H9	O89b	O89b		O8	17499/17499
**AR435**	CP029115.1	ST167	H9	O89b	O89b			17499/17499
**M217**	AP019189.1	ST167	H9	O89b	O89b			17499/17499
**CREC-532**	CP024830.1	ST167	H10	O89b	O89b			17499/17499
**CREC-629**	CP024815.1	ST167	H10	O89b	O89b			17499/17499
**Y5**	CP013483.1	ST167	H4	O89b	O89b			17499/17499
**AR_0162**	CP021683.1	ST167	H5	O89b	O89b			17499/17499
**AR_0114**	CP021732.1	ST617	H9	O89b	O89b			17497/17498
**MRSN346355**	CP018121.1	ST617	H9	O89b	O89b			17499/18274 (e)
**MRSN346638**	CP018115.1	ST617	H9	O89b	O89b			17499/18274 (e)
**MRSN346595**	CP018109.1	ST617	H9	O89b	O89b			17499/18274 (e)
**MRSN352231**	CP018103.1	ST617	H9	O89b	O89b			17499/18274 (e)
**5CRE51**	CP021175.1	ST617	H10	O89b	O89b			17498/17499
**SCEC020023**	CP025950.3	ST617	H10	O89b	O89b			17497/17499
**WCHEC005784**	CP028578.2	ST617	H10	O89b	O89b		O9	17499/18564 (e)
**675SK2**	CP027701.1	ST617	H10	O89b	O89b			17494/17497
**H8**	CP010172.1	ST617	H4	O89b	O89b			17497/17499
**1283**	CP023371.1	ST10	H10	O89b	O89b			17497/17499
**Ecol_422**	CP018962.1	ST10	H10	O89b	O89b			17497/17499
**DA33133**	CP029574.1	ST10	H10	O89b	O89b			17497/17499
**6409**	CP010371.1	ST10	H9	O89b	O89b	O9	O9	17497/17499 (f)
**26561**	CP027118.1	ST10	H9	O89b	O89b			17496/17497
**1223**	CP023383.1	ST744	H9	O89b	O89b			17495/17497
**EC590**	CP016182.2	ST744	H10	O89b	O89b			17493/17497
**W5-6**	CP032992.1	ST744	H10	O89b	O89b			17462/17512
**Ecol_AZ155**	CP019005.1	ST44	H4	O89b	O89b			11180/18274 (e)
**CH611_eco**	CP017980.1	ST4981	H9	O89b	O89b			17497/18273 (e)
**AR_0137**	CP021879.1	ST1284	H21	O89b	O89b			17497/17499

(a) O89b is most closely related to serotype O89 based *wzm* and *wzt* genes (93-94% sequence identity). (b) The *wzm* and *wzt*-containing gene cluster for O9 is located outside the O89b gene cluster, and O8 is a truncated *wzt* gene. (c) Homology: sequence identity/match length (bp). (d) Internal deletion compared to Sanji. (e) Internal insertion compared to Sanji. (f) Partial inversion compared to Sanji.
