# Supplementary material for: Comparative Genome Analysis of an Extensively Drug-Resistant Isolate of Avian Sequence Type 167 Escherichia coli Strain Sanji with Novel In Silico Serotype O89b:H9
Source: mSystems. 2019 Feb 26;4(1):e00242-18. doi: 10.1128/mSystems.00242-18 (PMC6392093; doi:10.1128/mSystems.00242-18)
Supplement: TABLE S2 [file mSystems.00242-18-st002.pdf]

Table S2. *E. coli* genomes carrying the 69.2-kb insertion.

***E. coli* strain**	**Sanji**	**CRE1540**	**MRY15-117**	**H8**	**14EC017**	**WCHEC4533**	**HB-Coli0**
Number of plasmids	6	4	2	3	3	4	3
Total Resistance Genes	32	36 (54) [a]	12	16	22 (24)	21	16
Chromosomal Res. Genes	6	18 (19)	6	6	6	6	6
Plasmid Res. Genes [b]	pSJ_255, 27<br>pSJ_82, 1	p1540-1, 2<br>p1540-2, 19 (23)<br>p1540-3, 12	pMRY15-117_1, 7<br>pMRY15-117_2, 1	plasmid B, 10	p14EC017a, 1<br>p14EC017b, 17	pCTXM15, 13<br>pNDM4, 2	unnamed1, 10

[a] Number indicates total non-redundent antibiotic resistance genes, number in parenthes indicate total count including replicates.

[b] Number following the plasmid names indicates the number of non-redundant antibiotic resistance genes on that plasmid.
