# Supplementary material for: Comparative Genome Analysis of an Extensively Drug-Resistant Isolate of Avian Sequence Type 167 Escherichia coli Strain Sanji with Novel In Silico Serotype O89b:H9
Source: mSystems. 2019 Feb 26;4(1):e00242-18. doi: 10.1128/mSystems.00242-18 (PMC6392093; doi:10.1128/mSystems.00242-18)
Supplement: TABLE S3 [file mSystems.00242-18-st003.pdf]

Table S3. Antibiotic resistance gene profile of pSJ_255-related IncHI2 plasmids.

Bacteria strain	***Serratia marcescens***	***Salmonella enterica Heidelberg***	***Escherichia coli APEC O1***	***Escherichia coli EC5207***	***Escherichia coli WCHEC050613***	***Escherichia coli SHP45***	***Escherichia coli XG-E1***	***Salmonella enterica Typhimurium GDS147***	***Escherichia coli WCHEC025943***	***Escherichia coli HS13-1***	***Escherichia coli Sanji***
IncHI2 plamid	**R478**	**pSH111_227**	**pAPEC-O1-R**	**pEC5207**	**pMCR**	**pHNSHP45-2**	**pXGE1mcr**	**pHXY0908**	**pMCR1_025943**	**pHS13-1**	**pSJ_255**
No. resistance genes	***6***	***8***	***6***	***4***	***23***	***25***	***25***	***27***	***27***	***30***	***27***
**aac(3)-Iva**					✔	✔	✔	✔	✔		✔
**aac(3)-Via**			✔								
**aac(3)***					✔	✔	✔	✔	✔		✔
**aac(6')Ib-cr**								✔		✔	✔
**aacA7**				✔							
**aadA1**			✔		✔	✔	✔	✔	✔	✔	✔
**aadA2**					✔	✔	✔	✔		✔	✔
**aph(3')-Ia**						✔	✔	✔	✔	✔	✔
**aph(3')-Ic**	✔										
**aph(3')-Iia**		✔									
**aph(4)-Ia**						✔	✔	✔	✔		✔
**aph(6)-Ic**		✔									
**arr-3**								✔		✔	✔
**blaCMY-2**				✔							
**blaCTX-M-14**					✔	✔	✔				
**blaCTX-M-65**									✔		
**blaOXA-1**								✔		✔	✔
**blaTEM-1B**									✔		
**ble**					✔	✔	✔	✔	✔	✔	
**catA1**	✔										
**catB3**								✔		✔	✔
**cmlA1**					✔	✔	✔	✔		✔	✔
**dfrA12**					✔	✔	✔	✔		✔	
**eamA**									✔	✔	
**estX**						✔	✔	✔			✔
**floR**						✔	✔	✔	✔	✔	✔
**fosA**					✔	✔	✔				
**glmM**					✔	✔	✔	✔			✔
**lnu(F)**									✔		
**mcr-1**					✔	✔	✔		✔	✔	
**mef(B)**										✔	
**mph(A)**					✔		✔		✔	✔	✔
**mphR**					✔		✔		✔	✔	✔
**mrx**					✔		✔		✔	✔	✔
**nimC/nimA**					✔	✔	✔	✔	✔	✔	
**oqxA**					✔	✔		✔	✔	✔	✔
**oqxB**					✔	✔		✔	✔	✔	✔
**oqxR**					✔	✔		✔	✔	✔	✔
**psp**						✔	✔	✔			✔
**qacE**					✔	✔	✔	✔	✔	✔	
**qacEd1**			✔	✔				✔		✔	✔
**qacI**					✔	✔	✔	✔		✔	✔
**qnrS2**										✔	
**strA**		✔							✔		
**strB**		✔							✔		
**sul1**			✔	✔	✔	✔	✔	✔	✔	✔	✔
**sul2**					✔	✔	✔	✔	✔		✔
**sul3**					✔	✔	✔	✔		✔	✔
**tet(A)**									✔	✔	
**tet(B)**	✔	✔									
**tet(C)**			✔								
**tet(M)**									✔	✔	✔
**tetC**	✔	✔									
**tetD**	✔	✔									
**tetR**									✔	✔	
**tetR(B)**	✔	✔									
**tetR(C)**			✔								
